# An Account of Diseases in an Eastern District of London from September 20, to October 20, 1809

**Published:** 1809-11

**Authors:** 


### *An Account of Diseases in an Eastern District of London from September 20, to October 20, 1809.*

| ACUTE DISEASES. | |
|---|---|
| Febris Intermittens - - | 1 |
| Catarrhus - - - - - - | 7 |
| Cynanche Tonsillaris - | 4 |
| Cholera - - - - - - | 5 |
| Pneumonia - - - - | 2 |
| Rheumatismus Acutus | 3 |
| **CHRONIC DISEASES.** | |
| Tussis - - - - - - - - | 15 |
| Dyspnœa - - - - - - | 7 |
| Tussis cum Dyspnœa - | 20 |
| Hydrothorax - - - - - | 4 |
| Hæmoptysis - - - - - | 3 |
| Diarrhœa - - - - - | 14 |
| Enterodynia - - - - - | 7 |
| Anasarca - - - - - - | 5 |
| Ascites - - - - - - - | 2 |
| Hæmorrhois - - - - - | 3 |
| Prolapsus Vaginæ - - | 1 |
| Prolapsus Ani - - - - | 1 |
| Dysuria - - - - - - - | 5 |
| Fluor Albus - - - - - | 7 |
| Menorrhagia - - - - - | 3 |
| Dysmenorrhœa - - - - | 2 |
| Hysteria - - - - - - - | 2 |
| Rheumatismus Chronicus | 7 |
| **PUERPERAL DISEASES.** | |
| Menorrhagia Lochialis - | 5 |
| Abcessus Mammæ - - | 2 |
| Rhagas Papillæ - - - | 4 |
| Dolores Post Partum - | 7 |
| **INFANTILE DISEASES.** | |
| Febris Mesenterica - - | 2 |
| Convulsio - - - - - - | 2 |
| Dentitio - - - - - - - | 1 |
| Tussis - - - - - - - | 4 |
| Aphthæ - - - - - - | 3 |

The different species of intestinal disease have lately prevailed to a considerable extent. Diarrhœas of the milder kind have been very frequent; but indeed so mild were the symptoms that they could hardly be said to constitute a disease. The pain was in general comparatively trifling, and the evacuations so gentle, that they might be considered as a salutary effort of the constitution, to relieve itself of something burdensome and offensive.

In some cases, the use of a gentle eccoprotic, and in others, the administration of a brisk cathartic, were found to be the most effectual mode of treatment.

Choler[illegible]

Cholera has been very frequent. In some instances, the disease appeared in its mildest form, and the evacuations by vomiting and purging, though sufficient to distinguish the disease, were no more than might, at any time, be occasioned by a slight accumulation of bile in the stomach and bowels. In other instances, however, the disease assumed a more unpleasant appearance.

Violent vomitings, and a considerable discharge from the bowels occurred, and these were attended with sharp pains in the course of the intestinal canal; with spasms in the lower extremities. In one instance, cold sweats and syncope occurred, as the precursors of a fatal termination, which took place in spite of every assistance that could be afforded.

The severity of cold, which was felt for a few days, has produced a premature introduction of some of those diseases which are peculiar to the winter. Cough and Catarrh, Dyspnœa and Peripneumonia, have been frequent at this early period. We may hope, however, that the return of a milder temperature, which has occurred within these few days, may at least suspend the aggravation of symptoms alarmingly dangerous.

